# A Constant Speed Changing Rate and Constant Turn Rate Model for Maneuvering Target Tracking

**DOI:** 10.3390/s140305239

**Published:** 2014-03-13

**Authors:** Guan Zhai, Huadong Meng, Xiqin Wang

**Affiliations:** Department of Electronic Engineering, Tsinghua University, Beijing 100084, China; E-Mails: zhaiguan@gmail.com (G.Z.); wangxq_ee@tsinghua.edu.cn (X.W.)

**Keywords:** dynamic model, constant speed changing rate and constant turn rate (CSCRCTR) model, maneuvering target tracking

## Abstract

This paper addresses the problem of modeling maneuvering target motion in tracking applications. A target trajectory can typically be divided into segments with different dynamic motion modes, such as a constant velocity motion, a constant acceleration motion or a constant turn rate motion. To integrate the different motion modes into a uniform model, a Constant Speed Changing Rate and Constant Turn Rate (CSCRCTR) model is proposed. A new state vector is defined, and the state transition function is derived. Based on the CSCRCTR model, we present a tracking algorithm using a particle filter. The performances of the CSCRCTR model, the uniform model (UM) and the interacting multiple model (IMM) for tracking a simulated maneuvering target are compared and show that the CSCRCTR model maintains a good consistency for different types of motions and achieves better accuracy than UM and IMM when maneuvers occur.

## 1. Introduction

In most tracking systems, the target motion is modeled as a system whose varying state makes a transition according to an underlying model or several switching models. More useful information about the target's state can be extracted from observations by using a more appropriate model for the target's motion. The selection of the proper model for applications of maneuvering target tracking is important and this topic has received much attention.

Many specific dynamic models of target motion have been developed for maneuvering target tracking. The simplest models for a maneuvering target are the white-noise acceleration model [1], which assumes the acceleration to be an independent process, and the constant-acceleration model [1], which assumes the acceleration to be a process with independent increments. The Singer acceleration model [2] assumes the acceleration to be a time-correlated stochastic process and lays the foundation for several other effective maneuver models, such as the mean-adaptive acceleration model [3] and the asymmetrically distributed normal acceleration model [4]. These models, in which the filters are uncoupled across the coordinates show poor performance for several typical maneuvers, such as turns. The constant turn rate (CT or CTR) model [5,6] replaces the acceleration with the turn rate. The CT model has been incorporated and extended in many other models [7,8]. Many filters and algorithms for maneuvering targets [1,5,9–12] are developed based on these specific dynamic models. The details of these models and more other dynamic models are illustrated in [9,13].

Each of the models described above performs well in specific scenarios, but there is no universally optimal model for all applications. The interacting multiple model (IMM) method [14–16] models the target motion as a hybrid system in which the state evolves according to a stochastic differential equation; the model jumps from one to another among a finite number of possible models according to a set of transition probabilities. The IMM algorithm constructed with a small number of models provides good estimations when the models cover the types of motion well. A bank of more models is required to cover various types of maneuvers, which reduces the effectiveness of the IMM due to the unnecessary competition between many non-matched models at any particular time [17].

The segmenting track identifier (STI) [18], which is a non-Bayesian tracker was proposed as an alternative approach for maneuvering target tracking. This data-driven estimator partitions a target trajectory into a sequence of segments and estimates the state parameters for each segment. The STI operates well in tracking a highly maneuvering target with unknown behavior such as a free-swimming fish, but does not perform as well in sophisticated weapon delivery, air traffic control or satellite surveillance systems in which the target motion is easier to predict. A key component of STI is to approximate one segment as a circular arc or as a straight line, which can also be treated as an arc with an infinite radius. Based on this idea, this paper proposes a constant speed changing rate and constant turn rate (CSCRCTR) model in which the trajectory in each frame is approximated as an arc. In many practical systems, the tangential force which makes a speed change and the normal force which makes a heading change are independent. By introducing the distances travelled during the last two frames and the heading angles at the last two sampling times to the state vector, the speed and the heading angle evolve independently in the state transition function. The CSCRCTR model matches well with a coordinated turn and a motion with a constant speed changing rate, which are the most common typical maneuvers.

This paper is organized as follows: the constant speed changing rate and constant turn rate (CSCRCTR) model for maneuvering target motion is proposed in Section 2. The tracking algorithm based on the CSCRCTR model, including the initialization and recursive filtering method, is described in Section 3. Section 4 presents a simulation of tracking a maneuvering target and compares the performances of the CSCRCTR model, the uniform motion (UM) model and the interacting multiple model (IMM). Section 5 summarizes concluding remarks.

## 2. Constant Speed Changing Rate and Constant Turn Rate model

In this study, we consider a target that is moving in a two-dimensional plane. In most traditional models, the state vector includes the position, the velocity, and the acceleration in both dimensions. The speed (magnitude of the velocity) and heading angle (direction of the velocity) are rarely included in the state. However, the direct result of a maneuver is a change of the speed or heading, which results in changes of the velocity and the acceleration. When the target starts to increase power, the resultant force in the movement direction causes the target to change speed; when the target starts to turn, the resultant force perpendicular to the movement direction makes the target change its heading angle.

Equations (1) and (2) are in the integral forms of the target motion:

(1) $$\nu_{t+T}=\nu_{t}+\int_{t}^{t+T}a_{\tau }d\tau$$

(2) $$\varphi_{t+T}=\varphi_{t}+\int_{t}^{t+h}\omega_{\tau }d\tau$$

where *ν_t_* and *φ_t_* are the speed and the heading angle of the target at time *t*, respectively, *a_τ_* and *ω_τ_* are the changing rate of the speed and the changing rate of the heading angle at time *τ*, respectively, *T* is the sampling interval. The parameters *ν_t_*, *φ_t_*, *a_τ_*, and *ω_τ_* are all scalars, but not vectors. The changing rate of the heading angle, *ω_τ_*, is the so-called turn rate.

Since observations from the sensors are usually available only at discrete times, the model is often designed in a discrete-time form. A system with a fixed sampling interval, which is very common in many applications, is considered in this paper. We also make two assumptions here:

(1) The speed changing rate is constant or nearly constant.
(2) The turn rate is constant or nearly constant.

The two assumptions are appropriate for both rectilinear motion with a uniform acceleration and a coordinated turn, which are two typical maneuvers. Obviously, the two assumptions both hold for a uniform motion. Based on these two assumptions, we have Equations (3) and (4):

(3) $$s_{c}-s_{p}\approx aT\approx {\mathrm{constant}}_{1}$$

(4) $$\varphi_{c}-\varphi_{p}\approx \omega T\approx {\mathrm{constant}}_{2}$$

where *s_c_* and *φ_c_* are the speed and heading angle at the current sampling time, respectively, *s_p_* and *φ_p_* are the speed and heading angle at the previous sampling time, respectively, *a* is the constant speed changing rate, and *ω* is the constant turn rate.

**Figure 1.**
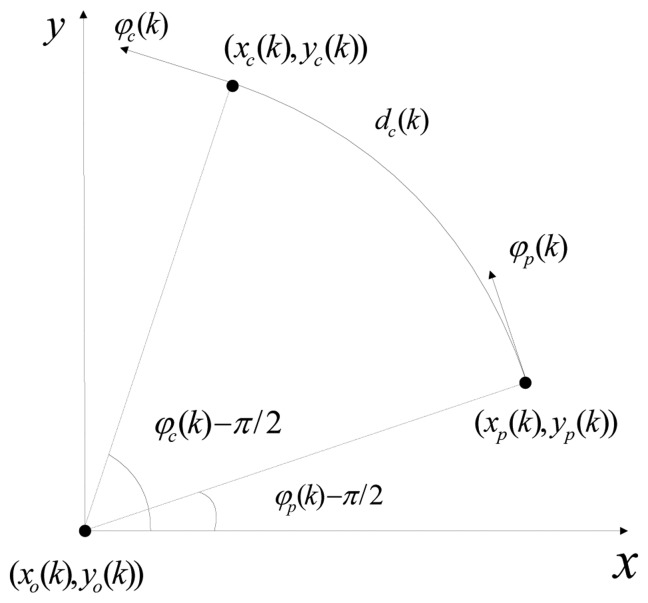
The geometric relationship between two neighboring samples.

As mentioned in Section 1, we approximate the trajectory between the previous sample and the current sample as an arc. The geometric relationship between the states at the two neighboring sampling times is shown in Figure 1.

For the *k*^th^ frame, (*x_p_* (*k*), *y_p_*(*k*)) and (*x_c_* (*k*), *y_c_*(*k*)) represent the positions at the previous sampling time and the current sampling time in a Cartesian coordinate system, respectively , (*x_o_* (*k*), *y_o_*(*k*)) represents the position of the center of the arc, and *d_c_*(*k*) represents the distance (not the displacement) during the current frame (the time between the previous sampling time and the current sampling time), which equals to the arc length. The geometric relationship between two neighboring samples can be written as Equations (5) and (6):

(5) $$x_{o}(k)=x_{c}(k)-r(k)•\sin\varphi_{c}(k)=x_{p}(k)-r(k)•\sin\varphi_{p}(k)$$

(6) $$y_{o}\left(k\right)=y_{c}\left(k\right)+r\left(k\right)•\cos\varphi_{c}\left(k\right)=y_{p}\left(k\right)+r\left(k\right)•\cos\varphi_{p}\left(k\right)$$

where *r*(*k*) represents the radius of the arc and can be written as (7):

(7) $$r(k)=\frac{d_{c}(k)}{\varphi_{c}(k)-\varphi_{p}(k)}$$

For the explicitness of the model construction and the simplicity of implementing the filter, *s_c_*, which is the speed at the current sampling time, and *s_p_*, the speed at the previous sampling time, are replaced by *d_c_*, the distance during the current frame, and *d_p_*, the distance during the previous frame. Equation (3) thus becomes (8):

(8) $$d_{c}-d_{p}\approx aT^{2}\approx {\mathrm{constant}}_{3}$$

Let *X*(*k*) = [*x*(*k*), *y*(*k*), *φ_c_*(*k*), *φ_p_*(*k*), *d_c_*(*k*), *d_p_*(*k*)]*^T^* denote the state vector at the *k*^th^ sampling time. By combining Equations (4)–(6) and (8), the transition function of the new proposed CSCRCTR model can be expressed as (9)–(14):

(9) $$\varphi_{c}(k)=2\varphi_{c}(k-1)-\varphi_{p}(k-1)+u(k-1)$$

(10) $$\varphi_{p}(k)=\varphi_{c}(k-1)$$

(11) $$d_{c}(k)=2d_{c}(k-1)-d_{p}(k-1)+\nu (k-1)$$

(12) $$d_{p}(k)=d_{c}(k-1)$$

(13) $$x\left(k\right)=x\left(k-1\right)+d_{c}\left(k\right)•\frac{\sin\varphi_{c}\left(k\right)-\sin\varphi_{p}\left(k\right)}{\varphi_{c}\left(k\right)-\varphi_{p}\left(k\right)}$$

(14) $$y\left(k\right)=y\left(k-1\right)-d_{c}\left(k\right)•\frac{\cos\varphi_{c}\left(k\right)-\cos\varphi_{p}\left(k\right)}{\varphi_{c}\left(k\right)-\varphi_{p}\left(k\right)}$$

The terms *u*(*k*−1) in (9) and *v*(*k*−1) in (11) represent the process noises, which are assumed to be two independent zero-mean Gaussian variables with variances of $\sigma_{\varphi }^{2}\left(k-1\right)$ and $\sigma_{d}^{2}\left(k-1\right)$, respectively.

## 3. Tracking Algorithm Based on the CSCRCTR Model

The algorithm based on the CSCRCTR model is described in this section. As shown in (13) and (14), the transition function is nonlinear. By inserting (9)–(12) into (13) and (14), we find that the additive noises in (9) and (11) due to the modeling errors or variations in the speed changing rate and turn rate are not additive to the entire state vector. A particle filter is used to implement the algorithm. The sensor model we consider in this paper is shown in a simple form as (15) and (16), and only the position data are obtained:

(15) Z(k)=H(k)X(k)+W(k)

(16) $$H(\text{k})={\left[\begin{array}{cccccc} 1 & 0 & 0 & 0 & 0 & 0\\ 0 & 1 & 0 & 0 & 0 & 0 \end{array}\right]}^{T}$$

*W*(k) is a zero-mean Gaussian white noise with a covariance Q(k) = *diag*(*σ*^2^, *σ*^2^). The first stage provides an initialization method and the second stage presents the filtering process.

### 3.1. Initialization

**Figure 2.**
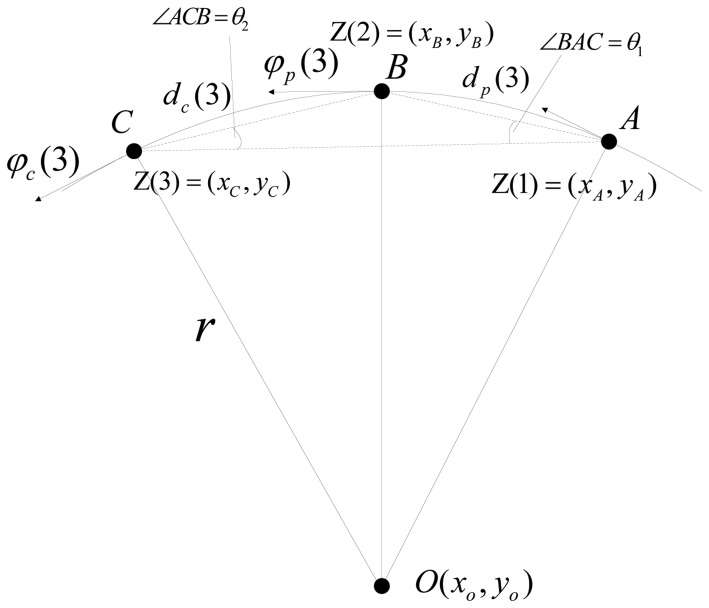
The geometrical relationship between the initial three observations.

At least three samplings of sensor observations are needed to initialize a track. As shown in Figure 2, the trajectory from the first observation to the third is still approximated as an arc. According to Heron's formula and the sine law, we can derive the initialization procedure using (17) to (27):

(17) $$\gamma =\left\|AB\right\|=\sqrt{{\left(x_{B}-x_{A}\right)}^{2}+{\left(y_{B}-y_{A}\right)}^{2}}$$

(18) $$\beta =\left\|AC\right\|=\sqrt{{\left(x_{C}-x_{A}\right)}^{2}+{\left(y_{C}-y_{A}\right)}^{2}}$$

(19) $$\alpha =\left\|BC\right\|=\sqrt{{\left(x_{C}-x_{B}\right)}^{2}+{\left(y_{C}-y_{B}\right)}^{2}}$$

(20) $$r=\frac{\alpha \beta \gamma }{\sqrt{\left(\alpha +\beta +\gamma \right)\left(\alpha +\beta -\gamma \right)\left(\alpha +\gamma -\beta \right)\left(\beta +\gamma -\alpha \right)}},\mathrm{if}\;\alpha +\gamma \neq \beta$$

(21) $$\theta_{1}=\{\begin{array}{ll} \arcsin\left(\frac{\gamma }{2r}\right), & \mathrm{if}\;\alpha +\gamma \neq \beta \\ 0, & \mathrm{if}\;\alpha +\gamma =\beta \end{array}$$

(22) $$\theta_{2}=\{\begin{array}{ll} \arcsin\left(\frac{\alpha }{2r}\right), & \mathrm{if}\;\alpha +\gamma \neq \beta \\ 0, & \mathrm{if}\;\alpha +\gamma =\beta \end{array}$$

(23) $$d_{c}(3)=\{\begin{array}{ll} 2r\cdot \theta_{1}, & \mathrm{if}\;\alpha +\gamma \neq \beta \\ \alpha , & \mathrm{if}\;\alpha +\gamma =\beta \end{array}$$

(24) $$d_{p}(3)=\{\begin{array}{ll} 2r\cdot \theta_{2}, & \mathrm{if}\;\alpha +\gamma \neq \beta \\ \gamma , & \mathrm{if}\;\alpha +\gamma =\beta \end{array}$$

(25) $$\eta_{BC}=\left\{ \begin{array}{l} \arctan\left(\frac{y_{C}-y_{B}}{x_{C}-x_{B}}\right),\mathrm{if}\;x_{C}-x_{B}>0\\ \arctan\left(\frac{y_{C}-y_{B}}{x_{C}-x_{B}}\right)+\pi ,\mathrm{if}\;x_{C}-x_{B}<0\\ \pi /2,\;\mathrm{if}\;x_{C}-x_{B}=0,y_{C}-y_{B}>0\\ -\pi /2,\;\mathrm{if}\;x_{C}-x_{B}=0,y_{C}-y_{B}<0 \end{array}\right.$$

(26) $$\varphi_{c}(3)=\left\{ \begin{array}{l} \eta_{BC}-\theta_{1},\mathrm{if the direction is clockwise}\\ \eta_{BC}+\theta_{1},\mathrm{others} \end{array}\right.$$

(27) $$\varphi_{p}(3)=\left\{ \begin{array}{l} \eta_{BC}+\theta_{1},\mathrm{if the direction is clockwise}\\ \eta_{BC}-\theta_{1},\mathrm{others} \end{array}\right.$$

The initial state estimation at the third sampling time is obtained by the derivation presented above. The results are shown in (23), (24) and (26)–(29):

(28) $$x(3)=x_{C}$$

(29) $$y(3)=y_{C}$$

The initial covariance of the state estimation can be written as (30):

(30) $$H(k)=P(3|3)=\left[\begin{array}{cccccc} \frac{8\sigma^{2}}{{\hat{s}}_{c}^{2}(3)} & -\frac{4\sigma^{2}}{{\hat{s}}_{c}^{2}(3)} & 0 & 0 & 0 & 0\\ -\frac{4\sigma^{2}}{{\hat{s}}_{c}^{2}(3)} & \frac{8\sigma^{2}}{{\hat{s}}_{c}^{2}(3)} & 0 & 0 & 0 & 0\\ 0 & 0 & 4\sigma^{2} & -2\sigma^{2} & 0 & 0\\ 0 & 0 & -2\sigma^{2} & 4\sigma^{2} & 0 & 0\\ 0 & 0 & 0 & 0 & \sigma^{2} & 0\\ 0 & 0 & 0 & 0 & 0 & \sigma^{2} \end{array}\right]$$

### 3.2. Recursive Filtering

The particle filter [10] has been widely used in nonlinear/non-Gaussian Bayesian estimation problems. Based on the principle of the particle filter, a complete cycle of recursive filtering is given in Table 1. Once a tracker is initialized, *N* particles can be drawn from the PDF of the estimated state which is assumed to be a Gaussian vector with a mean of the initial estimation and a covariance given by (30). For a new cycle of filtering, each particle sample is predicted according to the transition function of the CSCRCTR model. In the prediction step, the distances and the heading angles should be predicted first, followed by the positions. When a new observation comes, the mean, covariance and even the PDF can be estimated by calculating the likelihood of each sample and obtaining a normalized weight. To avoid the degeneracy phenomenon, the particles are resampled in every cycle.

## 4. Simulation Results

A simulated maneuvering target is tracked using the proposed CSCRCTR model. The CSCRCTR model is compared to two uniform motion (UM) models with different levels of process noise and an IMM composed of a UM model, a constant acceleration (CA) model and four different turn rate models.

### 4.1. The Scenario

**Figure 3.**
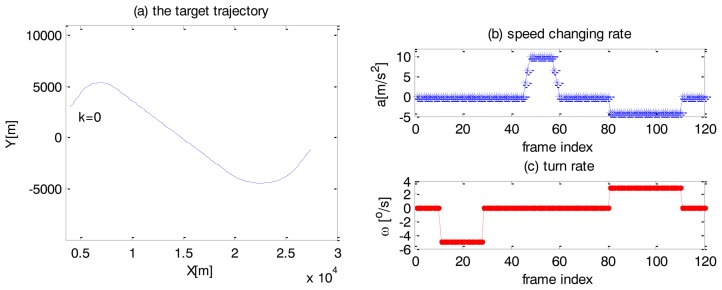
(**a**) Target trajectory (**b**) Speed changing rate of the target (**c**) Turn rate of the target.

The maneuvering target goes through seven stages, including uniform motions and several maneuvers. In the 1st stage (1st–10th frame), 3rd stage (29th–45th frame), 5th stage (61st–80th) and 7th stage (111th–120th frame), the target moves with a constant velocity. In the 2nd stage (11th–28th frame), it turns at a rate *ω* = −5°/*s*. In the 4th stage (46th–60th frame), it increases power with a speed changing rate which increases from *a* = 0*m*/*s*^2^ to *a* = 10*m*/*s*^2^ (46th–48th frame), then remains at *a* = 10*m*/*s*^2^ (49th–57th frame) and decreases from *a* = 10*m*/*s*^2^ to *a* = 0*m*/*s*^2^ (58th–60th frame). In the 6th stage (81st–110th frame), it simultaneously turns with a rate *ω* = 3°/*s* and decelerates at a speed changing rate *a* = −4*m*/*s*^2^. The trajectory and the indicators of the maneuvers, such as the speed changing rate and the turn rate, are shown in Figure 3.

The initial position of the target is [4,000 m, 3,000 m], the speed is 200 m/s and the heading angle is 53.13°. The sensor can only provide the Cartesian coordinates:

(31) $$Z={\left[x,y\right]}^{T}+W$$

where *W* is a zero-mean Gaussian white noise with a covariance Q = *diag*(*σ*^2^, *σ*^2^). The parameter *σ* is assumed to be 40 m, so the coordinate-combined raw measurement error is approximately 57 m. The sampling interval *T* = 1*s*.

### 4.2. Parameters of the Different Models

The UM model [1] is a constant velocity model. In a 2D tracking application, the state vector is defined as *X* = [*x*, *ẋ*, *y*, *ẏ*,], and it follows the simplest constant velocity model. The process noise, which is a design parameter, can be used to account for turbulence or an acceleration, *etc.* UM is a linear model and can be implemented using a Kalman filter. The process noise is a normally zero-mean Gaussian white noise with a covariance of Q in the form of (32).

(32) $$Q=\left[\begin{array}{cccc} \sigma_{x}^{2}T^{4}/4 & \sigma_{x}^{2}T^{3}/2 & 0 & 0\\ \sigma_{x}^{2}T^{3}/2 & \sigma_{x}^{2}T^{2} & 0 & 0\\ 0 & 0 & \sigma_{y}^{2}T^{4} & \sigma_{y}^{2}T^{3}/2\\ 0 & 0 & \sigma_{y}^{2}T^{3}/2 & \sigma_{y}^{2}T^{2} \end{array}\right]$$

In this paper, a low level of *Q* with $\sigma_{x}^{2}=\sigma_{y}^{2}=150$ and a high level of *Q* with $\sigma_{x}^{2}=\sigma_{y}^{2}=400$ are selected to compare with the CSCRCTR model. The target is also tracked using an IMM algorithm, which includes a nearly constant velocity model (UM), a nearly constant acceleration (CA) model and four coordinated turn (CT) models with different turn rates. Since the turn rate is known in each model, the models are linear. The transition matrix is written as (33):

(33) $$\phi_{w}=\left[\begin{array}{cccc} 1 & \frac{\sin\omega T}{\omega } & 0 & \frac{1-\cos\omega T}{\omega }\\ 0 & \cos\omega T & 0 & \sin\omega T\\ 0 & \frac{1-\cos\omega T}{\omega } & 1 & \frac{\sin\omega T}{\omega }\\ 0 & \sin\omega T & 0 & \cos\omega T \end{array}\right]$$

The form of the process noise is similar to (32), and the process noise $\sqrt{q}$ is selected to be 5, which is of the same level as a noise $\sigma_{x}^{2}=\sigma_{y}^{2}=25$ in (32). The turn rates of these four models are −6°/*s*, −3°/*s*, 3°/*s* and 6°/*s*. The process noise in the UM model is assumed to be very small ( $\sigma_{x}^{2}=\sigma_{y}^{2}=1$) to make an accurate estimation during the uniform motion. To track additional maneuvers, a CA model is added to the set of models. The process noise in the CA model is assumed to be $\sigma_{x}^{2}=\sigma_{y}^{2}=64$ to track the maneuvers other than a coordinated turn. The basic idea of this IMM algorithm is based on [13], which provides additional details of this method. Although the performance of the IMM does not rely on the transition probability of the models, we provide the numbers used in (34) in the simulation:

(34) $$p_{ij}=\left[\begin{array}{cccccc} 0.9 & 0.02 & 0.02 & 0.02 & 0.02 & 0.02\\ 0.06 & 0.9 & 0.01 & 0.01 & 0.01 & 0.01\\ 0.06 & 0.01 & 0.9 & 0.01 & 0.01 & 0.01\\ 0.06 & 0.01 & 0.01 & 0.9 & 0.01 & 0.01\\ 0.06 & 0.01 & 0.01 & 0.01 & 0.9 & 0.01\\ 0.06 & 0.01 & 0.01 & 0.01 & 0.01 & 0.9 \end{array}\right]$$

### 4.3. Results

**Figure 4.**
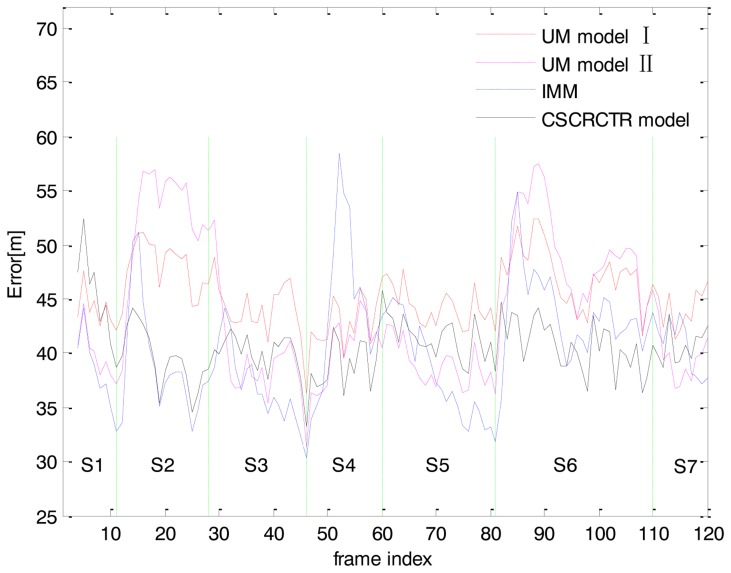
RMS position errors.

The results are obtained based on 100 independent Monte Carlo simulations. The RMS position errors for the two UM models, the IMM and the CSCRCTR model are shown in Figure 4. The UM model I refers to the UM model with a high level of process noise and the UM model II refers to the model with a low level of process noise. Each model has its advantages and disadvantages. The UM model II works very well in the steady state, but it deteriorates sharply when a maneuver occurs. Although the UM model I has the flexibility to adapt to different physical behaviors of the target, it cannot obtain an accurate estimation for any type of motion. The RMS position errors of UM model I are greater than those of the proposed CSCRCTR model at almost all sampling times.

The CSCRCTR model can estimates the heading angle well and provide good position estimations during the coordinated turn between the 11th and 28th frames. The IMM performs slightly better because the turn is perfectly matched with one of the multiple models. For the rectilinear motion with a constant acceleration between the 49th and 57th frames, the CSCRCTR model performs much better than the IMM. Even when the speed changing rate is not a constant (Frames 46–49), the CSCRCTR model gives better estimations because it reacts faster to the accelerating tendency. For a compound maneuver in which the target decelerates and turns at the same time, the CSCRCTR model provides the most accurate estimation. However, when the target is in the uniform motion mode (Frame 81–110), there is a certain loss in the steady-state performance because we use an arc to approximate the trajectory, which is actually a straight line.

When the target maneuvers in the 2nd, 4th and 6th stage, the CSCRCTR model reacts quickly and converges with a high rate. The IMM algorithm is considered to have rapid response to maneuvers, but the CSCRCTR model is more sensitive to changes of motion modes.

If we transform the state estimated to Equation (35) based on the assumption of a constant speed changing rate, we can estimate the speed:

(35) $${\hat{s}}_{c}(\text{k})=\frac{3d_{c}(\text{k})-d_{p}(\text{k})}{2T}$$

**Figure 5.**
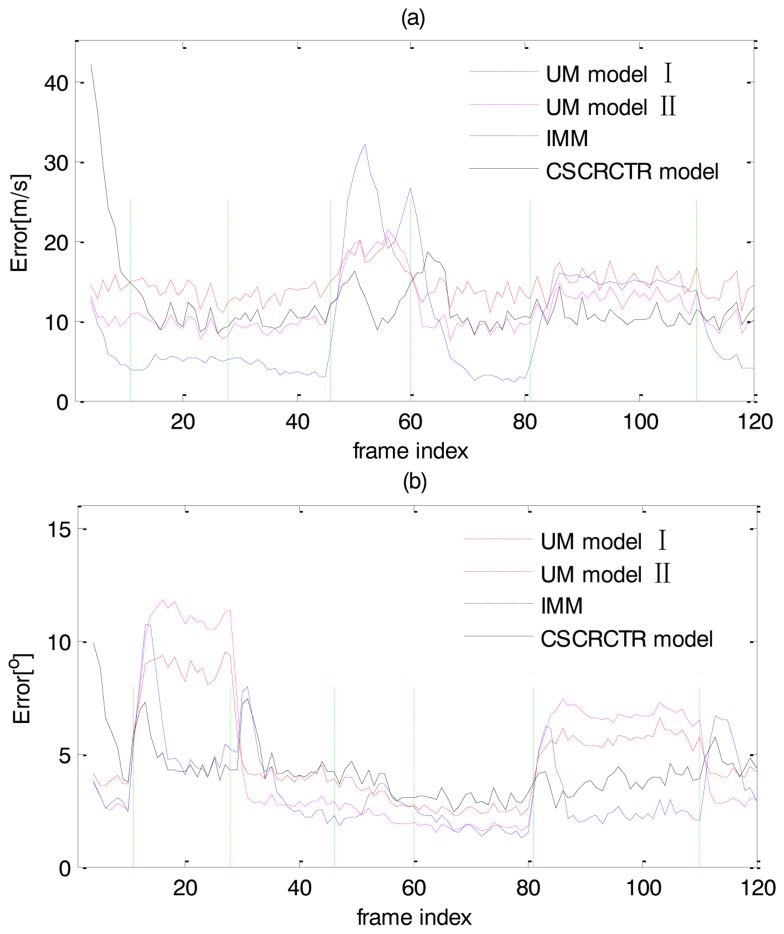
(**a**) RMS speed errors; (**b**) RMS heading angle errors.

In the two UM models and the IMM, the speed and heading angle can also be calculated based on the estimation of [*ẋ*, *ẏ*]*^T^*. The RMS speed and heading angle errors are presented in Figure 5. The results also reveal that the CSCRCTR model has a good performance when the target maneuvers. Specifically, the CSCRCTR evaluates the speed well when the target accelerates and the heading angle well when the target turns.

The average tracking errors of each model for each maneuver are listed in Table 2. The highest errors for each segment of motion are marked in italics and the lowest errors are marked in bold. Table 2 gives a similar conclusion as the results shown in Figures 4 and 5 and provides more details. Every segment of motion is separated into the beginning stage (“B”), which includes the first five frames, and the stable stage (“S”), which includes the other frames. When the target begins a coordinated turn (Frames 11–28), the IMM and the CSCRCTR model both react quickly to the maneuver and provide good position estimations. During the turn, the CSCRCTR model estimates the heading angle well, while the IMM estimates the speed accurately. During a constant acceleration motion (Frames 49–57), the CSCRCTR model outperforms the other models in estimating the position and speed because the accelerating behavior matches the model (Equation (11)). When the target is in the uniform motion (Frames 61–80) and there are no speed and heading changes, the errors of the CSCRCTR model are slightly higher because it overestimates the change of distance or heading angle caused by measurement noise. For a compound maneuver in which the target decelerates and turns at the same time (Frames 81–110), the CSCRCTR model describes both behaviors well and provides the best estimations. The peak RMS position estimation errors for UM I, UM II, IMM and CSCRCTR are 49.1 m, 54.4 m, 48.9 m and 41.8 m, respectively. The CSCRCTR model shows improvements of approximately 14% over IMM, 15% over UM I, and 23% over UM II. As shown in Table 2, the CSCRCTR model never achieves the highest position errors among the four models in any stage and shows great stability. Although it does not yield the lowest errors during the uniform motion, CSCRCTR shows good comprehensive performance for different types of motions, including typical and compound maneuvers.

## 5. Conclusions

In most traditional models, the velocities and the accelerations in different dimensions are coupled according to the target motion. A new state vector, which includes the positions, the distances travelled in the current and previous frames, and the heading angles of the current and previous sampling times, is defined in this paper. We proposed the CSCRCTR model for tracking a maneuvering target based on the assumptions of constant speed changing rate and constant turn rate by approximating the trajectory between two adjacent samplings as an arc. The model can be adapted to different types of maneuvers as it treats the speed and the heading angle independently. Due to the obvious nonlinearity of the model, a particle filter is used to implement the tracking algorithm.

Compared to the UM model and the IMM, the CSCRCTR model reacts faster to a maneuver and obtains more accurate estimations during the maneuvering period. The CSCRCTR model yields an improvement of approximately 14% in the peak RMS position estimation error for all types of motions over the IMM and more significant improvements over the UM models. It provides a more accurate speed estimation for an accelerating target and a more accurate heading angle estimation for a turning target. The CSCRCTR model is adaptable to different types of maneuvers and achieves a good balance between stability and maneuverability.

## Figures and Tables

**Table 1.** Summary of the recursive filtering algorithm.

Initialization	Set of particles {*X͂_i_*(3):*i* = 1,2,…,*N*}, each with a weight 1/*N* Posterior distribution: $p\left(X\left(3\right)|Z\left(3\right)\right)=\sum_{i=1}^{N}\frac{1}{N}\delta \left(X\left(3\right)-{\tilde{X}}_{i}\left(3\right)\right)$
	Set *k* = 3

for each cycle, *k* = *k*+1			

Prediction	Generate *N* samples drawn from the distribution of *u*(*k*−1), *ν*(*k*−1) {*u_i_*(*k*−1), *ν_i_*(*k*−1):*i* = 1,2,…,*N*} for *i* = 1: *N* $\varphi_{ci}^{*}\left(k\right)=2\varphi_{ci}\left(k-1\right)-\varphi_{pi}\left(k-1\right)+u_{i}\left(k-1\right)$ $\varphi_{pi}^{*}\left(k\right)=\varphi_{ci}\left(k-1\right)$ $d_{ci}^{*}\left(k\right)=2d_{ci}\left(k-1\right)-d_{pi}\left(k-1\right)+\nu_{i}\left(k-1\right)$ $d_{pi}^{*}\left(k\right)=d_{ci}\left(k-1\right)$ $x_{i}^{*}\left(k\right)=x_{i}\left(k-1\right)+d_{ci}^{*}\left(k\right)•\frac{\sin\varphi_{ci}^{*}\left(k\right)-\sin\varphi_{pi}^{*}\left(k\right)}{\varphi_{ci}^{*}\left(k\right)-\varphi_{pi}^{*}\left(k\right)}$ $x_{i}^{*}\left(k\right)=x_{i}\left(k-1\right)+d_{ci}^{*}\left(k\right)•\frac{\cos\varphi_{ci}^{*}\left(k\right)-\cos\varphi_{pi}^{*}\left(k\right)}{\varphi_{ci}^{*}\left(k\right)-\varphi_{pi}^{*}\left(k\right)}$
Filtering	Weight calculation: $q_{i}\left(k\right)=\frac{1}{2\pi \sigma^{2}}\exp\left(-\frac{{\left(Z_{1}\left(k\right)-x_{i}^{*}\left(k\right)\right)}^{2}+{\left(Z_{2}\left(k\right)-y_{i}^{*}\left(k\right)\right)}^{2}}{2\sigma^{2}}\right),i=1:N$ Weight normalization: $q_{i}\left(k\right)=\frac{q_{i}\left(k\right)}{\overset{N}{\underset{j=1}{\text{∑}}}q_{j}\left(k\right)},i=1:N$ State estimation: $X\left(k|k\right)=\sum_{i=1}^{N}q_{i}\left(k\right)X_{i}^{*}\left(k\right)$ Covariance of the state estimation: $\text{P}\left(k|k\right)\sum_{i=1}^{N}q_{i}\left(k\right)\left(X_{i}^{*}\left(k\right)-X\left(k|k\right)\right){\left(X_{i}^{*}\left(k\right)-X\left(k|k\right)\right)}^{T}$ Posterior distribution: $p\left(X\left(k\right)|Z\left(k\right)\right)=\sum_{i=1}^{N}q_{i}\left(k\right)\delta \left(X\left(k\right)-X_{i}^{*}\left(k\right)\right)$

Updated particles	Set of particles { $X_{i}^{*}\left(k\right):i=1,2,\ldots ,N$}, each with a weight *q_i_*(*k*)

Resampling	Resample *N* times from the posterior distribution and obtain { $X_{i}^{*}\left(k\right):i=1,2,\ldots ,N$}, each with a weight 1/*N*

**Table 2.** Average tracking errors.

**Motion type**		**Coordinated Turn**		**Acceleration**		**Simultaneously Deceleration and Turn**		**Uniform Motion**	

Stage of motion *		**B**	**S**	**B**	**S**	**B**	**S**	**B**	**S**

**Frame index**		**11–15**	**16–28**	**49–53**	**54–57**	**81–85**	**86–110**	**61–65**	**66–80**
Average RMS position error[m]	UM I	*46.9*	49.1	42.3	44.0	*47.9*	47.0	*46.1*	*43.7*
	UM II	44.9	*54.4*	39.7	42.9	46.5	*49.3*	**41.5**	38.2
	IMM	42.5	**37.7**	*48.9*	*47.3*	44.7	43.4	44.1	**36.4**
	CSCRCTR	**41.8**	38.6	**38.9**	**38.7**	**41.7**	**40.3**	42.2	40.9

Average RMS speed error [m/s]	UM I	*14.7*	*12.9*	18.1	18.9	*13.9*	*15.6*	13.5	*13.6*
	UM II	10.6	9.2	17.6	18.8	12.2	12.8	**10.7**	9.7
	IMM	**4.4**	**4.9**	*24.2*	*23.8*	**11.0**	15.1	*14.8*	**4.0**
	CSCRCTR	12.5	10.2	**13.1**	**10.3**	10.7	**10.5**	14.4	10.2

Average heading angle error [°]	UM I	8.2	8.8	*3.9*	*3.1*	4.8	5.6	*2.8*	*2.6*
	UM II	*9.6*	*11.2*	2.6	**2.2**	*5.8*	*6.9*	**2.0**	**1.8**
	IMM	8.8	5.0	**2.1**	3.2	5.2	2.5	2.3	1.7
	CSCRCTR	**6.4**	**4.4**	3.0	2.8	**3.6**	**3.8**	2.3	2.4


* Each period of motion is divided into the beginning stage (B) and the stable stage (S).
